# Influence of guar gum and chitosan enriched with lemon peel essential oil coatings on the quality of pears

**DOI:** 10.1002/fsn3.2851

**Published:** 2022-03-29

**Authors:** Ayesha Iftikhar, Abdur Rehman, Muhammad Usman, Ahmad Ali, Muhammad Mushtaq Ahmad, Qayyum Shehzad, Hina Fatim, Arshad Mehmood, Abdul Moiz, Muhammad Asim Shabbir, Muhammad Faisal Manzoor, Azhari Siddeeg

**Affiliations:** ^1^ 66724 National Institute of Food Science and Technology University of Agriculture Faisalabad Faisalabad Pakistan; ^2^ Department of Agricultural Environmental and Food Sciences (DiAAA) University of Molise Campobasso Italy; ^3^ 66374 State Key Laboratory of Food Science and Technology School of Food Science and Technology Jiangnan University Wuxi Jiangsu China; ^4^ 58276 Beijing Advance Innovation Center for Food Nutrition and Human Health School of Food and Health Beijing Technology and Business University Beijing China; ^5^ Department of Food Science and Technology Riphah International University Faisalabad Faisalabad Pakistan; ^6^ Section of Chemical and Food Engineering Department of Industrial Engineering University of Salerno Fisciano Italy; ^7^ 12676 School of Food and Biological Engineering Jiangsu University Zhenjiang Jiangsu China; ^8^ 119098 Department of Food Engineering and Technology Faculty of Engineering and Technology University of Gezira Wad Medani Sudan

**Keywords:** chitosan, edible coatings, guar gum, lemon peel essential oil, pear

## Abstract

Pear is a typically climacteric fruit and highly perishable with a low shelf life owing to extreme metabolic activity after harvesting. The present study aimed to reduce weight loss and improve the firmness of pear during storage. The lemon peel essential oil (LPEO) has gained considerable attention due to being the richest source of bioactive compounds that behaved as a natural antioxidant agent, being cost‐effective, and being generally recognized as safe. Edible coatings equipped with a natural antioxidant agent and renewable biopolymers have gained more research fame owing to their involvement in the direction of biodegradability and food safety. In this work, edible skin coating materials (ESCMs) embedded by chitosan (1%) and guar gum (2%) were fabricated, and afterward, five concentrations of LPEO (1, 1.5, 2, 2.5, and 3.0%) were incorporated individually into the ESCMs. Findings revealed that LPEO–ESCMs significantly reduced the weight loss and improved the firmness of pear up to 45 days of storage at 4 ± 2°C. Furthermore, the LPEO–ESCMs have enhanced the antioxidant capacity, antibacterial efficiency, and malondialdehyde level of pear during storage time. It was concluded that 3% of LPEO–ESCMs improved the overall acceptability of pear fruits. Taken together, the novel insights of guar gum and chitosan‐based ESCMs entrapped with LPEO will remain a subject of research interest for researchers in the future.

## Graphical abstract

Postharvest losses of pears are a key problem for exporters and traders. The weight loss and firmness of pears have been reduced during storage, while LPEO–ESCMs delayed it. LPEO–ESCMs also improved the ascorbic acid, TPC, and antioxidant profile of pears. The 3% concentration of LPEO–ESCMs showed good sensory attributes. It shows an innovation to minimize the post‐harvest losses of climacteric fruits.

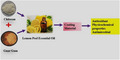

## 1 INTRODUCTION

The consumers’ interest regarding the consumption of fruits has been increasing extensively because these are well renowned to be one of the significant pillars of a healthy diet (Maringgal et al., 2020). However, the postharvest losses resulting in the deterioration of the quality and quantity of the fruits are key problems that are faced in the modern era (Nair et al., 2020). These losses are likely due to poor handling and storage or unsuitable packaging, promoting microbial, and fungal infections. As a novel plant, pear fruit (*Pyrus communis* L) is a member of the *Rosaceae* family and has become very popular among the consumers due to its good taste, thin peel, crisp flesh, and low caloric contents, and an excellent source of vitamin C and dietary fiber (Lindo‐García, Giné‐Bordonaba, et al., 2020). Many researchers revealed that the pear fruit is vulnerable to enzymatic browning and oxidative instability, leading to spoilage during cold storage (Lindo‐García, Larrigaudière, et al., 2020; Sharma & Rao, 2015). After harvesting, the respiration rate and ripening process led to significant changes in the color, firmness, flavor, acidity, and total soluble solids of pear fruits (Lindo‐García, Muñoz, et al., 2020). Therefore, some persuasive mechanisms have been developed to preserve pear fruits without affecting the nutritive value and sensory attributes (Lindo‐García et al., 2019). Over the years, several storage techniques have been established to prolong the lifetime of fruits, including modified atmosphere packaging (MAP) and controlled atmosphere (CA). The MAP and CA storage research on CO_2_ and O_2_ injury have investigated to increase ethylene production rate and flavor problem due to anaerobic respiration (Lindo‐García et al., 2019). Thus, an edible coating is a modified atmosphere technique that has exhibited excellent findings for enhancing the quality of the fruits (Guimaraes et al., 2018). Evidence‐based results exhibited that edible coating could function as a barrier on the fruit's surface, extending the shelf life by modifying the internal gas atmosphere, slowing down the ripening process, and reducing water losses (Maringgal et al., 2020). Recommended renewable biopolymers that are being used in the development of edible coatings are polysaccharides, lipids, and proteins (Garavand et al., 2020). Among the aforementioned biopolymers, polysaccharides are known to be potent candidates used to improve the quality of fresh fruits (Barclay et al., 2019). As polysaccharides have numerous benefits over artificial polymers due to their biodegradable, biocompatible, and renewable characteristics and compliance to environmentally friendly modifications (Rehman et al., 2020; Usman et al., 2021; Zhao et al., 2020). Moreover, edible coatings prepared from polysaccharide sources not only enhance the fruit quality but also provide strong protection against undesirable factors such as heat, moisture contents, and enzymatic degradation. Guar gum is a galactomannan comprising of a mannose [(1 → 4)‐linked β‐D‐mannopyranose] backbone with galactose side groups [(1 → 6)‐linked α‐D‐galactopyranose]. It is obtained from the endosperm of the plant *Cyamopsis tetragonoloba* which belongs to the *Leguminosae* family. Chitosan is also one of the considerable natural polymers that have utilized for the shelf‐life extension of fruits under a range of different storage conditions (Garavand et al., 2020). The abovementioned organic polymers have commonly been used to inhibit the growth of different food‐related microbes such as *Escherichia coli*, *Bacillus subtilis*, and *Staphylococcus aureus* by retarding their growth (Jawad et al., 2017). Moreover, these have been suggested for film formation and coatings due to high water‐soluble capacity, easy solubility in organic acids, high molecular weight, and long polymeric chains (Li et al., 2016). Recently, chitosan‐based coatings enriched with ascorbic acid and procyanidin markedly improved the antioxidant activity of mango (up to 24 days at 15 ± 2°C with 85%–90% relative humidity [RH]) and fresh blueberries (at 4°C for 14 days), respectively (Mannozzi et al., 2018). Likewise, pullulan, calcium chloride, and chitosan have been used to preserve the whole pear for 30 days at 0 ± 1°C, by enhancing the total antioxidant activity significantly. Similarly, authors have found that the best pear coating is 2% chitosan and 1% pullulan as it provides an insulating barrier to the surface of the pear and improves the environment around the fruit. Furthermore, the researchers have preserved mango (Silva et al., 2017) and guava (Silva et al., 2018) fruits with chitosan solution, which have slowed down the water losses, respiratory rate, climacteric peak, firmness quality, and skin color by interjecting the degradation of chlorophyll up to 20 days and 96 h, respectively, at 25 ± 2°C and 85 ± 3% RH. Conclusively, the results indicated that the edible chitosan coating effectively improved fruit quality by starch degradation and mitochondrial respiration reduction. Gum and ginseng extract coatings were prepared in 2018 to preserve the quality of sweetened cherry for up to 8 days, at 20°C (Dong & Wang, 2018). Findings revealed that the organic polymer coating can delay the production of malondialdehyde. Furthermore, the mechanism also markedly eradicates weight loss, slows down respiration rate, delays the changes in ascorbic acid and anthocyanins, and maintains the membrane integrity throughout the storage period. However, efforts have been made to introduce novel natural protecting materials, for example, essential oils were used to maintain fresh fruits' safety and quality (Azarakhsh et al., 2014). An earlier research investigated the effects of a minimum inhibitory concentration of pectin‐ and alginate‐based edible coatings enhanced with eugenol and citral or their mixture (Guerreiro et al., 2016). According to that study, both pectin‐and alginate‐based coatings preserved fresh‐cut 'Bravo de' Esmolfe’ apples. However, the insight of chitosan‐ and guar gum‐based edible coatings and their antimicrobial and antioxidant agent like lemon peel essential oil (LPEO) remains to be investigated. Therefore, the present study was designed to inhibit the growth of microbes and provides a suitable environment around pear fruits using ESCMs enriched with LPEO; however, the storability of the pear was explored.

## 2 MATERIALS AND METHODS

The healthy and undamaged pear fruits were harvested in early August and stored at 6–8°C with 85 ± 5% RH in the cold storage chamber. A skinning machine was used for peeling. The fruit was processed in two parts: peel and pulp, leading to freezing by liquid nitrogen at −80°C. Before the experiment, the samples were grounded (IKA^®^‐WERKE MF 10 basic S1 (1000 W, 50/60 Hz) microfine grinder drive equipped with a 0.5 mm sieve was used for cryogenic grinding) into powder form. The chemicals with 95% purity used in the experiments were purchased from Sigma‐Aldrich Pvt. Ltd. and Merck‐Millipore Pvt. Ltd.

### 2.1 Proximate analysis of fresh pear

The sample of dried pear was examined through the approved methods (AOAC, 2006) to calculate mineral content (method no. 985‐30), crude protein (method no. 984‐13), crude fiber (method no. 32‐10), crude fat (method no. 30‐10), ash (method no. 942‐05), and moisture (method no. 934‐01). Moreover, the total sugar content of fresh pear was evaluated as described by Mahapatra et al. (2012).

### 2.2 Extraction of LPEO

The lemon was obtained from Ayub Agriculture Research Institute (AARI), Faisalabad, Pakistan. In the round‐bottomed flask, 100 g lemon peel was pulverized and steeped in water. LPEO were extracted by hydrodistillation (400 ml of distilled water) for up to 4 h with an industrial Clavenger device at the boiling range of water and atmospheric pressure. The Clevenger device consisted of a 1000‐ml round‐bottomed flask (Isolab), reflux condenser (Norm Cam), and a volatile determination tube. Ground glass connector was connected to every object. The essential oil is measured on Clevenger equipment after the extraction time has passed. Until the quality assessment analysis, the LPEO was placed at refrigeration temperature (4 ± 1°C). The following equation was used to compute the production of lemon oil: $Y=\;\frac{V}{W}\;\times 100$; where *Y* is the production of LPEO in percentage (v/w), *V* is the LPEO acquired (ml), and *W* is the quantity of lemon peels (g). The findings were obtained for the various extraction times of 60, 120, 180, and 240 min for lemon peel. The average yield of LPEO was 3.5%; however, it depends on the extraction conditions such as extraction time, water‐to‐material ratio, and extraction power (Dao et al., 2021).

### 2.3 Physicochemical analysis of LPEO

The LPEO was examined for specific gravity (method no. 10A‐25), refractive index (method no. 7‐95), free fatty acid (FFA) (method no. 5a‐40), saponification value (method no. 3c‐91), and p‐anisidine value (method no. 18‐ 90) by the method as presented in AOAC (2006). The peroxide value of LPEO was determined as described by Xu et al. (2013).

### 2.4 Preparation of ESCMs solutions enriched with LPEO

Edible skin coating materials, adding the LPEO, were formulated as defined by Azevedo et al. (2014). Moreover, the preparation of chitosan solution (100 ml) was formed by liquefying chitosan (w/v) in a 1.5% acetic acid (0.5 ml acetic acid/100 ml deionized H_2_O) solution at 25°C for 3.5 h and incorporating 1.28% glycerol (w/v) to the mixture while heating the solution on a hot plate. In the case of guar gum solution (100 ml), 2% of guar gum (w/v) and 0.64% of glycerol (w/v) were added into distilled water and heated the solution into a water bath up to 70°C for 30 min as described by Mehyar et al. (2011), with minor modification. After cooling (25°C), the mixture was incorporated into the chitosan solution and mixed well until it was homogeneous. Then, the solution was autoclaved at 121°C for 15 min in a glass bottle. Lastly, the LPEO was dissolved in ESCMs solution, and the concentration of guar gum, chitosan, and LPEO used in each treatment has exhibited in Table 1.

**TABLE 1:** Treatment plan for pear fruit coating

Treatment	LPEO (%)	Chitosan (%)	Guar gum (%)
T_0_	–	–	–
T_1_	1.0	1.0	2.0
T_2_	1.5	1.0	2.0
T_3_	2.0	1.0	2.0
T_4_	2.5	1.0	2.0
T_5_	3.0	1.0	2.0

Abbreviation: LPEO, Lemon peel essential oil.

### 2.5 Analysis of LPEO–ESCMs solution

Titratable acidity (TA), pH, and viscosity have a particular impact on fruits' quality. The pH of the LPEO–ESCMs solution was determined using a digital meter (Mettler FE20, Mettler‐Toledo). The pH meter was calibrated through buffer solutions before utilization for experiments. The acidity of LPEO–ESCMs solution was measured using the following formula: TA (%) = (*V* × 0.1 N NaOH × 0.009 × 100/*m*); where '*V*' is the titer volume of NaOH, and '*m*' is the weight of LPEO–ESCMs solution (ml). The viscosity of the LPEO–ESCMs solution was evaluated as described by Lin and Zhao (2007).

### 2.6 LPEO–ESCMs treatments

The whole fresh pear was sorted into comprehensive arbitrary groups, cleaned from dirt, debris, or any other particles by 50 ppm sodium hypochlorite solution, and then washed with distilled water. Afterward, the washed fresh pear was dried at room temperature through hygienic air by adopting the methodology of de Aquino et al. (2015). The pear was coated with chitosan and guar gum (ESCMs) encapsulating five different concentrations (1, 1.5, 2, 2.5, and 3.0%) of LPEO, while the control sample was placed in the distilled water (Table 1). After dipping the pears, they were dried on the nylon sheet. Moreover, 1920 healthy and uniform pears were picked and divided into six treatments, and every treatment contained 320 pears. There were three replications in treatment, and each replication comprises of 106 pears. The corrugated three‐ply fiberboard boxes were utilized to pack the pears using 5% perforation with paper lining and then the packed pears boxes were kept into a cold storage chamber (at 4 ± 2°C and up to 95% RH for 45 days). The antioxidant capacity, microbiological analysis, and physicochemical changes in pear fruits were explored at 15, 30, and 45 days of storage.

### 2.7 pH of coated pear fruits

The digital pH meter was used to measure the pH value of coated and uncoated pear as documented by Tiwari et al. (2008). Before evaluation, the digital pH meter was calibrated with two buffer solutions: acidic solution (with pH 4.0) and basic solution (with pH 7.0).

### 2.8 Total soluble solids and organic acid content of coated pear fruits

Total soluble solid and malic acid contents were obtained from fresh‐frozen tissue of pear as presented by Nath et al. (2012). TSS content was measured by diluting 2 g of pear flesh tissue in 62.5% (v/v) aqueous methanol solvent and kept in a thermostatic water bath for 10 min at 60°C and blending the mixture using vortex every 3 min to avoid layering. Afterward, the sample was centrifuged at 24,000 *g* for 12 min at 18°C. The supernatants of every treatment were obtained and utilized for enzyme‐attached spectrophotometric measurement of sucrose (β‐fructokinase), fructose, and glucose (hexokinase/phosphoglucose and isomerase) via commercial kits (BioSystem SA). In malic acid measurement, 2 g of frozen flesh tissue was dissolved in 5 ml distilled water, the treatments were slightly shaken for 8 min at 25°C, and then, centrifuged at 24,000 *g* for 6 min at 18°C. The resultant supernatant was found and used for enzyme‐coupled spectrophotometric measurement (L‐malate dehydrogenase) of malic acid through commercial kits (BioSystem SA).

### 2.9 Weight loss of coated pear fruits

The determination of weight loss was an important parameter to check the pear quality during the entire storage. The weight loss (%) was calculated using an MS6002TS balance (Mettler‐Toledo GmbH) by difference between the initial weight of pear and final weight of pear (at every storage interval) as per the method described by Kaur et al. (2019).

$$\mathrm{WL}\;\left(\%\right)=\;\frac{M1\;‐M2}{M1}\;\times 100$$

### 2.10 Firmness of coated pear fruits

The pear fruits firmness was calculated from the flesh of the pear, after removal of a thin part of the skin, at the equatorial area of the pear through a Fruit Texture Analyzer (Güss Manufacture Ltd) with an 8‐mm‐diameter probe as presented by Dong and Wang (2018). The trigger edge was fixed at 1 N, while the calculating speed and distance were 10 mm/s and 8.9 mm, respectively.

### 2.11 Antioxidant capacity and malondialdehyde (MDA) contents

The antioxidant activity of control and coated pears was studied by using the 2,2‐diphenyl‐1‐picryl‐hydrazyle (DPPH) test as per the procedure of Usman et al. (2020). Each sample (1 g) of pear fruit extract was mixed in 3.0 ml of ethanol, and centrifuged at 4°C for 15 min with the 12,000 *g* rpm, for analyzing DPPH. Then, the 3.9 ml DPPH–ethanol mixer, 0.1 ml supernatant, and 0.009 ml distilled water were vigorously mixed and shaken before keeping it in darkness for 30 min. Afterward, the absorbance was determined at a wavelength of 515 nm against a blank reference of ethanol without DPPH using a spectrophotometer (U‐2900, Hitachi). The antioxidant activity was measured by calculating the percentage of DPPH radical scavenging ability using an equation as mentioned below:

$$\text{DPPH}\;\text{activity}\;\left(\%\right)\;=\;\left(A_{c}‐\;A_{s}\right)/A_{c}\times \;100.$$

*A*_c_ and *A*_s_ represent the absorbance of the control (a mixture having 0.009 ml distilled water instead of sample supernatant) and sample, respectively, after 30 min of incubation.

Malondialdehyde is a method to estimate the lipid oxidation progress, which was performed as presented by Martínez‐Solano et al. (2005) using the thiobarbituric acid reactive substrates (TBARS). The absorbance was measured using a spectrophotometer (U‐2900, Hitachi) with a wavelength of 532 nm. The outcomes were exhibited as nmol/kg/s.

### 2.12 Microbiological analysis

The total viable count (TVC) of both the control sample and coated pear was estimated using a normal saline solution (NSS) and nutrient agar media by following the procedure of Sharma and Rao (2015). The NSS was prepared using an 8.5 g/L of sodium chloride (NaCl) and diluted in the melted sample and autoclaved at 121°C for 15 min. The nutrient agar media were made using 100 ml of distilled water dissolved in 2.8 g of agar, leading to autoclaved at 121°C for 15 min. The six test tubes were labeled (i.e., 10^–1^, 10^–2^, 10^–3^, 10^–4^, 10^–5^, and 10^–6^) and the 9 ml of NSS has been poured into each test tubes. The 1 ml sample was poured into the first test tube (10 ^–1^), and then the 1 ml sample was poured into the second test tube. A total of six dilutions were made in the same way. The nutrient agar media were poured into the Petri dish to solidify. Then, 1 ml sample from each dilution with a sterilized pipette was plated on Petri plates using a steak plate method. An inoculate was spread over an agar plate by using a sterilized glass spreader and was incubated at 37°C for 24 h. The colonies were counted using a colony counter in the Petri dishes; the average number of colony sizes was ranging from 30 to 300. TVC was calculated after the multiplication of the obtained count with reciprocal of selected dilution and expressed as colony forming units (log cfu/g).

TVC(logcfu/g)=Averagenumberofcolonies×Dilutionfactor/volumefactor.

### 2.13 Sensory estimation

The fruit's sensory score was measured by using 9‐point hedonic scale (1–9), where 1 = extremely unwanted, 2 = very much unwanted, 3 = moderately desirable, 4 = slightly undesirable, 5 = neither desirable nor undesirable, 6 = slight desirable, 7 = moderately desirable, 8 = very much desirable, and 9 = extremely desirable for five appropriate quality features (e.g., color, texture, odor, taste, and overall acceptability). The judges (9 women and 9 men, between the age group of 30 and 55 years) were selected from NIFST and Department of Post‐Harvest Technology, AARI, based on previous knowledge, interest, experience, and availability as per the selection method of Cliff and Toivonen (2017) with little change. Pear fruits have been taken from the cold storage chamber and cut into two slices, serving the judges for sensory evaluation. The slices were chosen from each pear to utilize in every duplicate measurement. The Panelists picked two slices of every treatment, while they were free to pick another sample. Coded treatments were shown in arbitrary order on the white tray for ensuring sincerity. The radar chats have been made by using the score of different quality attributes in Microsoft Excel 2013.

### 2.14 Data analysis

The research was performed out in 2020 and 2021 and arranged with a completely randomized design (factorial) using three replicates. Results were joined for the homogeneity of variance of 2 years of studies. Outcomes were evaluated by two‐way analysis of variance (ANOVA) and averages were differentiated through the least significant test. The variable findings among treatments were considered statistically significant at *p* ≤ .05 level of significance with a statistical software SAS (version 9.3 for Windows). Study outcomes were shown as mean ± standard error.

## 3 RESULTS AND DISCUSSIONS

### 3.1 Compositional analysis of fresh pear

The moisture, crude protein, crude fat, crude fiber, ash, total sugar, and mineral contents were found to be 80.41 ± 1.45% 5.4 ± 0.65%, 1.02 ± 0.13%, 4.9 ± 0.67%, 0.77 ± 0.10%, 6.3 ± 0.59, and 1.2 ± 0.23%, respectively (Table 2). The results were in agreement with the findings of Palma et al. (2015). The minor difference in the results could be due to the different growth conditions, harvesting practices, and postharvest conditions, or variations in climate, maturity, and soil conditions.

**TABLE 2:** Proximate composition of pear fruit

Parameters	Results (%)
Moisture	80.41 ± 1.45
Crude protein	6.4 ± 0.65
Mineral content	1.2 ± 0.23
Crude fiber	4.9 ± 0.67
Ash content	0.77 ± 0.10
Total sugar	6.3 ± 0.59
Fat	1.02 ± 0.13

### 3.2 Physicochemical analysis of LPEO

Lemon peel essential oil is a rich source of bioactive compounds such as tannins, phenolic acids, anthocyanins, and flavonoids. Thus, it has excellent antioxidant capacity and nutritional value that can improve the quality of final products. However, the physicochemical analysis of LPEO has been designed before application with ESCMs. Therefore, it is essential to evaluate the FFAs, refractive index, peroxide value, p‐anisidine value, saponification value, and the specific gravity of LPEO (Table 3). The FFAs were formed by the breakdown of triglycerides through hydrolysis (Shewfelt & Del Rosario, 2000). The FFAs were exclusively prone to oxidation, which resulted in off‐flavor of essential oil during storage. The LPEO was carried by 1.89 ± 0.2% FFAs value closely related to the findings of Giwa et al. (2018), which have 1.91 ± 0.3% FFAs. Refractive index (RI) has been performed to evaluate the possible chances of rancidity development. It is important to mention that the higher RI is directly proportional to oils' spoilage and oil‐based products. Typically, RI is an optical parameter, which has been used to analyze the light rays traversing through material or sample (Uysal et al., 2011). RI of LPEO was found to be 1.43 ± 0.02 measured in this study. Olabanji et al. (2016) have shown the RI of LPEO of about 1.46 ± 0.03. The p‐anisidine value was determined to check the secondary oxidation in foods, mostly 2, 4‐alkadienals and 2‐alkenals, which were generated due to the hydroperoxide. The LPEO have been subjected to p‐anisidine value in the range 3.36 ± 0.67, which was very close, as determined by Olabanji et al. (2016). The LPEO have exhibited the p‐anisidine value of up to 3.39 ± 0.85. Saponification value has been calculated to know about the chain length of molecule and types of glycerides in essential oil. The higher saponification value has indicated the presence of shorter‐chain fatty acids in major proportion. In our findings, the saponification value of LPEO was 40 ± 2.45 mg KOH/g, which is similar to the study of Giwa et al. (2018), revealing the saponification value of about 43.71 mg KOH/g. The saponification value of LPEO was markedly low, which makes it the best choice for application as ESCMs. The low density or specific gravity is essential to form the separate layer on the surface of fruits. Essential oils are most volatile and have low density compared with water (Ferhat et al., 2006). The specific gravity of LPEO was found to be 0.82 ± 0.023. The specific gravity of LPEO was found to be 0.82 ± 0.023. The findings of specific gravity (0.84) were very close, as presented by Ahmad et al. (2006). The peroxide index is the most common parameter used to predict lipid peroxidation (Kamal et al., 2011). The LPEO was exhibited in the range 5.56 ± 0.57 mEqO_2_/kg, which was similar to the results of Olabanji et al. (2016) who observed the peroxide value of LPEO of about 5.25 mEqO_2_/kg.

**TABLE 3:** Physicochemical analysis of Lemon peel essential oil

Parameters	Results
Free fatty acid (oleic acid %)	1.89 ± 0.23
Refractive index	1.43 ± 0.01
p‐Anisidine value	3.36 ± 0.67
Saponification value (mg KOH/g)	40.17 ± 2.45
Specific gravity	0.828 ± 0.0023
Peroxide value (mEqO_2_/kg)	5.56 ± 0.57

### 3.3 Physicochemical analysis of ESCMs

The TA and pH of ESCMs solution were found in the range 0.22 ± 0.004% (malic acid) and 3.6, respectively (Table 4), which are closely related to the results of Wu et al. (2016). Moreover, the thickness of ESCMs was noted at 0.093 ± 0.008 mm. The thickness of the pear was similar to the study of Dhumal et al. (2019).

**TABLE 4:** Analysis of edible skin coating materials

Parameter	Results
Titratable acidity (malic acid %)	0.22 ± 0.04
pH	3.6 ± 0.2
Viscosity (m^2^/s)	0.09 ± 0.08

### 3.4 Physicochemical analysis of coated pear fruits

#### 3.4.1 pH and Titratable acidity

**FIGURE 1:**
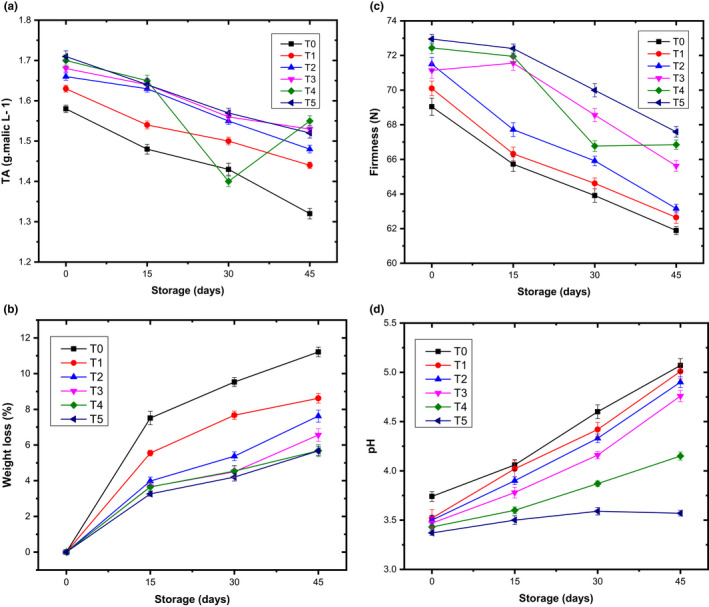
Variation in titratable acidity (TA) (a), weight loss (b), pear firmness (c), and pH (d) level of pear fruit during controlled storage in relation to multiple treatments of lemon peel essential oil‐edible skin coating materials (LPEO–ESCMs). Vertical bars represent ±*SD* of means for four replicates (*p* ≤ .05)

Evidence‐based results have shown that the average pH of coated pear was increased from 3.50 to 4.58 at 0 to 45 days of storage interval, respectively. However, the pH value has to be controlled using a different treatment of LPEO–ESCMs. It was noted that 3% of LPEO prominently reduced the pH of pear fruit as expressed in Figure 1d. Wang et al. (2007) exhibited similar results regarding the apple's pH value and prevented the increase in pH value during the entire storage. The TA measurement is an important quality indicator for fruits and vegetables and harmed climacteric fruit during storage. In climacteric fruits, like pear, malic acid is a major organic acid and considered TA of pear. During the entire storage, the LPEO–ESCMs reduced malic acid production from 1.66 g malic/L (at 0 day) to 1.47 g malic/L (at 45 days) as described in Figure 1a. Contrarily, the pear's TA content treated with LPEO–ESCMs exhibited a substantial difference against control pear on the termination of sampling. TA's degradation was prompt in untreated pear compared with LPEO–ESCMs‐treated pear from 0 to 45 days of storage. The reason behind this reduction in malic acid could be the conversion of organic acid into sugars or its utilization during the process of respiration (Grande‐Tovar et al., 2018).

#### 3.4.2 Weight loss

Weight loss in fresh fruits and vegetables is a critical attribute regarding economic losses. The weight loss in pear fruits is due to variation in metabolic activities, such as respiration rate, the activity of fruit softening enzymes, and transpiration of water during the entire storage time (Cheng et al., 2009). As mentioned above, the study agreed with Singh et al. (2009) and Zheng et al. (2017) for plum and kiwi fruits, respectively. In our study, a substantial increase in weight loss was noticed in pear fruits with the increase in the cold storage duration from 0 to 45 days regardless of the given treatments as shown in Figure 1b. However, the LPEO–ESCMs reduced the weight loss compared with untreated pear fruits during the whole storage. The lowest weight loss (3.28%) was noted in the coated pear with 3% of LPEO–ESCMs, whereas the highest weight loss (7.06%) was observed in the untreated pear. This study's results were similar to the findings of Medeiros et al. (2012) that reduced the weight loss in pear fruits by the application of calcium‐based coating material. A correlation study found that the relationship between mass loss and fruit firmness was the opposite (Adhikary et al., 2021). Additionally, edible coatings are believed to reduce weight loss owing to their semipermeable membrane barrier properties (Gol et al., 2013; Valero et al., 2013), as proved in a variety of fruits, such as sweet cherry, pepper, litchi, peach, and apricot (Ayranci & Tunc, 2003). Furthermore, discrepancies in the ability to inhibit weight loss have been linked to the varying water vapor permeability of the polysaccharides employed to formulate the edible coating (Vargas et al., 2008). As per certain experts (Serrano et al., 2008), adding glycerol into the coating as a plasticizer improved the weight retention of particular fruits. This could be because fresh‐cut fruit is considerably more prone to water loss than whole fruit, and the polysaccharides seem to be more permeable to water‐ than lipid‐based coatings.

#### 3.4.3 Pear firmness

The level of fruit maturity and rate of ripening process can be easily determined by assessing fruit's firmness. Firmness is directly connected to fruit texture, consumer acceptance in esteem to crispiness, and storability of fruit. Findings have revealed that the LPEO–ESCMs noticeably affected the firmness of pear fruits at each storage interval. The firmness of pear fruits was reduced from 71.12% to 64.62% regardless of LPEO–ESCMs treatments during the whole storage time (Figure 1c). However, the pear softening rate was much lower in LPEO–ESCMs compared to the untreated pear. The variation in pear firmness to variable LPEO treatments was practically significant and high in 45‐day storage ahead in contrast to 30 days, where 1%, 1.5%, 2%, 2.5%, and 3% LPEO exhibited similar results. The outcomes were comparable as per the discussion of Dave et al. (2017). Finally, we can conclude that fruit firmness had an inverse relationship with storability. It is also established that the fruit firmness could be triggered due to the activity of fruit‐softening enzymes. Therefore, the fruit firmness can be maintained by reducing the activity of *polygalacturonase*, *cellulose, hemicellulose,* and *pectin methylesterase* (Kaur et al., 2019). Contrarily, the softening of fresh‐cut apples was noted after application of edible coatings with essential oils, and caused the acid hydrolysis of pectic acid in fresh‐cut apple cell wall due to the low pH of film‐forming solutions of edible coatings containing essential oils (Raybaudi‐Massilia et al., 2008). Additionally, it has been claimed that such textural degradation may be produced by the essential oils penetrating the fruit's cell tissue causing structural alterations (Salvia‐Trujillo et al., 2015).

#### 3.4.4 Total soluble solid contents

**FIGURE 2:**
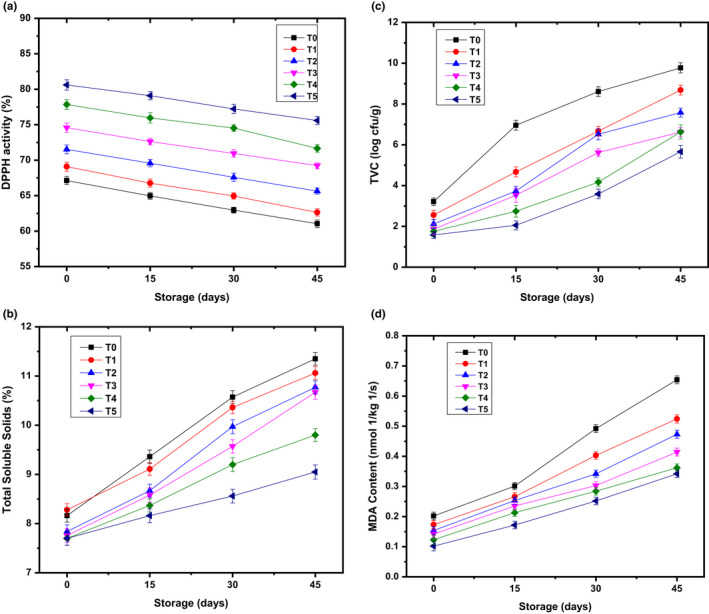
Variation in DPPH (a), total soluble solids (b), total viable count (TVC) (c), and MDA (d) content of pear fruit during controlled storage in relation to multiple treatments of lemon peel essential oil‐edible skin coating materials (LPEO–ESCMs). Vertical bars represent ±*SD* of means for four replicates (*p* ≤ .05)

The Total soluble solid contents (TSSC) is the best eating quality of pear fruits and consumer acceptability during storage. The TSSC has been increased in fruits using carbohydrate synthesis, which accelerated the ripening of fruits (Nath et al., 2012). In this study, the TSSC contents augmented throughout the storage irrespective of treatments with the rapid rate in untreated pear fruits compared with LPEO–ESCMs pear as expressed in Figure 2b. Steadily, TSSC increased until 45 days for all the variable concentrations of LPEO. Even so, in this storage period, the untreated pear also demonstrated higher TSSC contents than a treated pear. Moreover, the lowest TSSC contents were seen in coated pear with 3% LPEO–ESCMs, while the highest TSSC was recorded in a control sample of about 8.44% and 9.86%, respectively. Findings unveiled that the LPEO–ESCMs were practically important to work as a barrier against the increase in TSSC and slow down the ripening process rate. At 45 days of storage, the decline in TSSC might be due to the consumption of sugars during metabolic activities during storage. The outcomes are similar to the study conducted by Champa et al. (2014). Dave et al. (2017) have added 0.98% olive oil and 0.20% potassium sorbate in protein‐based edible coatings to preserve the pear fruits for several weeks and improved the TSSC of fresh fruits with the loss in fruits’ mass and progress in respiration rate; however, it has an inverse relationship with the fruit firmness (Adhikary et al., 2021).

### 3.5 Antioxidant capacity

The free radicals' theory of aging is very common in the scientific literature, which has been produced due to oxidative stress. Secondly, the degradation of polyphenols in pear fruits may had occurred due to the direct oxidation by polyphenols oxidases along with different oxidation. The findings of the current research explained the effect of LPEO, which has been a rich source of bioactive compounds. Therefore, the LPEO improved the antioxidant activity of treated pear fruits compared with untreated pear fruits throughout the storage period. Quality evaluation of antioxidants is very important; thus, a DPPH assay was performed to evaluate the treated pear fruits with LPEO–ESCMs. Results showed that coated pear antioxidant capacity was reduced irrespective of different treatments of LPEO–ESCMs at the entire pear fruits storability (Figure 2a). On the first day of storage, the pear fruits exhibited the highest free radical scavenging activity (RSA %), which was 73.45%, while gradually reduced to 67.63% at 45 days of storage. However, the highest RSA % was noted in 3% LPEO–ESCMs of about 78.18% followed by the treatments 1%, 1.5%, 2%, and 2.5%, which were near to 65.84%, 68.59%, 71.85%, and 75.00%, respectively. Contrarily, the untreated pear fruits have shown the lowest RSA of up to 64.02%. The findings were closely associated with the research conducted by Jin et al. (2012), which enhanced the antioxidant capacity of Chinese bayberry fruit using different EO, including linalool, perillaldehyde, cinnamaldehyde, and carvacrol.

### 3.6 MDA level

The off‐flavor or rancid flavor is a prevalent challenge in the postharvest produces during storage. Recently, Lindo‐García et al. (2019) have proven that the softening and ripening of 'Blanquilla' pear fruits are linked to oxidative stress, leading to higher MDA levels and increasing the climacteric rate. Moreover, they were also illustrated that the ethylene production in off‐tree ripened 'Conference' pear due to the softening and increase in lipid peroxidation (MDA level). The abovementioned studies indicated that the membrane lipid peroxidation processes are critical attributes accelerating the ripening of pear fruits. The present study results exhibited that the MDA level of treated pear fruits was increased regardless of varying concentrations of LPEO–ESCMs in a defined storage time as described in Figure 2d. Moreover, the MDA level was higher at 45 days, which was found to be 0.45 nmol/kg/s, whereas the lowest MDA value observed at the first day of storage was up to 0.15 nmol/kg/s. Although, the LPEO is practically reduce the MDA level of treated pear fruits. The findings showed that the MDA level was noticed by 0.22 nmol/kg/s for treated pear fruits with 3% LPEO–ESCMs than 0.41 nmol/kg/s for untreated pear fruits. Our findings are in line with Li et al. (2010), who reported that MDA content increase as prolong the storage period, but, the pear fruits treated with ESCMs could slow down the rate to MDA level compared with untreated pear fruits.

### 3.7 Microbiological analysis

Fresh and cold storage fruits and vegetables are prone to microbial contamination. The detection of spoilage in fresh fruits and vegetables might be possible when the microbial level reached above 7 logs CFU/g (Sharma & Rao, 2015). The microbial load of treated pear fruits was increased irrespective of varying treatments from 2.01 to 7.49 log CFU/g at 0 to 45 days of storage (Figure 2c). However, the microbial count of uncoated pear fruits was higher than coated pear fruits. Moreover, the TVC was observed as 7.14 log CFU/g and 3.22 log CFU/g for untreated pear fruit (0% LPEO–ESCMs) and treated pear fruit with 3% LPEO–ESCMs, respectively. The findings depicted that the rate of bacterial growth in coated pear fruits was minimum than uncoated pear fruits. The research indicates that the primary goal of incorporating essential oils and/or their components into edible coatings is to serve as antioxidant and antibacterial regulators. As per Azarakhsh et al. (2014), an alginate‐based coating optimized formulation with lemongrass oil drastically decreased total microbes, yeast, and mold counts in coated fresh‐cut pineapple samples when compared to the control group, and the same effect was observed for 'Fuji' apples infused with carnauba–shellac wax comprising of lemongrass oil (Jo et al., 2014). According to Raybaudi‐Massilia et al. (2008), an alginate coating alone did not significantly lower psychrophilic aerobic bacteria, yeast, or mold counts on fresh‐cut 'Fuji' apples. It can be concluded that the LPEO has an antibacterial effect and is a potential candidate to reduce the microbial load of fruits throughout the storage period.

### 3.8 Sensory evaluation

**FIGURE 3:**
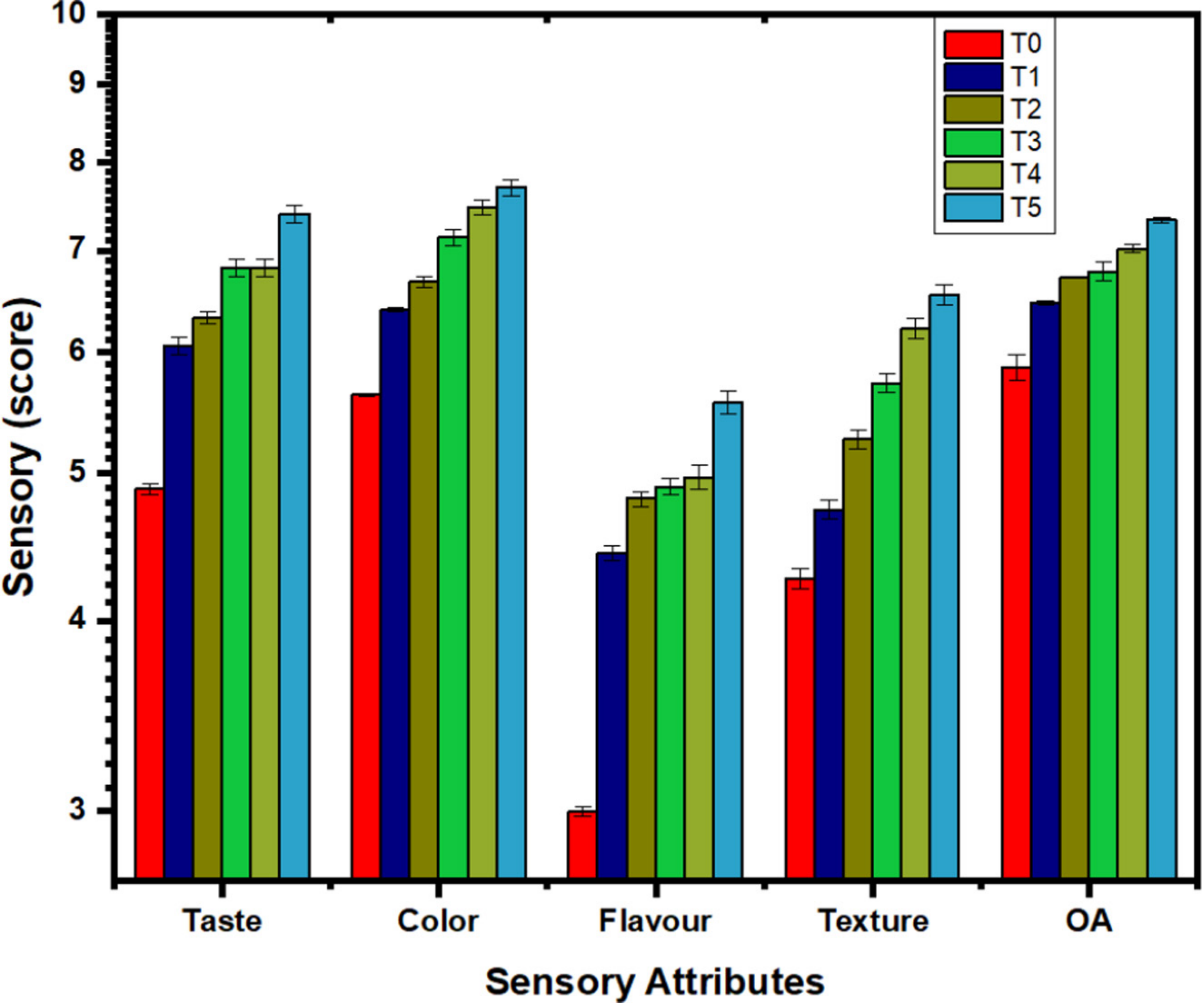
Effect of lemon peel essential oil‐edible skin coating materials’ (LPEO–ESCMs) treatments on taste, color, flavor, texture, and overall acceptability (OA) score (on 9‐point Hedonic scale) of pear fruit during 0, 15, 30, and 45 days of storage

It was quite interesting that the effect of LPEO–ESCMs regarding sensorial characteristics was noticeable in climacteric fruit rather than in non‐climacteric ones (Adhikary et al., 2021). The inclusive sensory attributes are imperative for the measurement of fruits' storability. The overall sensory quality of pear was decreased during each storage interval irrespective of all treatments as shown in Figure 3. However, the color, taste, texture, flavor, and overall acceptability scores were improved using 3% LPEO–ESCMs, which followed close linearity with the treated pear with 1%, 1.5%, 2%, and 2.5% LPEO of up to 45 days of storage at refrigerated temperature. The control pear revealed the maximum overall acceptability on the first day of storage which decreased steadily. On the contrary, the inclusion of lemon grass for up to 0.3% (w/v) in alginate‐based coating did not impact on the sensory attribute of treated fresh‐cut pineapples (Azarakhsh et al., 2014). However, the incorporation of 0.5% (w/v) lemongrass markedly improved the sensory properties of coated fruits. In another study, the sensory characteristics have also improved by chitosan‐based coatings enriched with essential oil (Perdones et al., 2012). This meant the addition of essential oil can modify the sensory profile of fresh‐cut fruits during storage, but the concentration of essential oil is important to induce the overall differences in the sensory attributes, and 3% LPEO concentration changed the general sensory quality attributes.

## 4 CONCLUSION

This research illustrated the effect of varying LPEO–ESCMs concentrations on the storability behavior of pear fruits at 4 ± 2°C. In the recent work, 3% LPEO–ESCMs concentration improved most of the ripening‐related changes, including soluble solids content, antioxidant activity, firmness score, and TA level. Besides, it also reduced weight loss, microbial load, and respiration rate of treated pear fruits than untreated pear. Therefore, based on these results, it was concluded that the LPEO–ESCMs utilization is a great leap forward to alleviate storability and retain the quality of pear fruits. However, the best combination of coatings was incorporated with the high concentration of essential oil (3%), which is not adequate for the larger scale, considering that the costs of essential oils are expensive. Keeping in view, the research gaps were identified on a correlation analysis of pear fruits’ enzymes activity and pear fruits’ firmness and mass loss. Furthermore, more information is required on a biotechnological intervention about the gene expression by the application of LPEO–ESCMs.

## CONFLICT OF INTEREST

The authors declare no conflict of interests.

## COMPETING INTEREST

The authors confirm that they have no known competing financial interests or personal relationships that could have appeared to influence the manuscript.

## CONSENT TO PARTICIPATE

All authors extend consent to participate as coauthors.

## Data Availability

The dataset supporting the conclusions of this article is included within the article.
